# Did Our Species Evolve in Subdivided Populations across Africa, and Why Does It Matter?

**DOI:** 10.1016/j.tree.2018.05.005

**Published:** 2018-08

**Authors:** Eleanor M.L. Scerri, Mark G. Thomas, Andrea Manica, Philipp Gunz, Jay T. Stock, Chris Stringer, Matt Grove, Huw S. Groucutt, Axel Timmermann, G. Philip Rightmire, Francesco d’Errico, Christian A. Tryon, Nick A. Drake, Alison S. Brooks, Robin W. Dennell, Richard Durbin, Brenna M. Henn, Julia Lee-Thorp, Peter deMenocal, Michael D. Petraglia, Jessica C. Thompson, Aylwyn Scally, Lounès Chikhi

**Affiliations:** 1 School of Archaeology, University of Oxford, South Parks Road, Oxford OX1 3TG, UK; 2 Department of Archaeology, Max Planck Institute for the Science of Human History, Kahlaische Street 10, D-07745 Jena, Germany; 3 Research Department of Genetics, Evolution and Environment, and University College London (UCL) Genetics Institute, University College London, Gower Street, London WC1E 6BT, UK; 4 Department of Zoology, University of Cambridge, Cambridge CB2 3EJ, UK; 5 Department of Human Evolution, Max Planck Institute for Evolutionary Anthropology, Deutscher Platz 6, D-04103 Leipzig, Germany; 6 Department of Archaeology, University of Cambridge, Pembroke Street, Cambridge, CB2 3DZ, UK; 7 Department of Anthropology, University of Western Ontario, London, ON, N6A 3K7, Canada; 8 Department of Earth Sciences, The Natural History Museum, Cromwell Road, London SW7 5BD, UK; 9 Department of Archaeology, Classics and Egyptology, University of Liverpool, 12-14 Abercromby Square, Liverpool L69 7WZ, UK; 10 Center for Climate Physics, Institute for Basic Science, Busan, South Korea; 11 Pusan National University, Busan, South Korea; 12 Department of Human Evolutionary Biology, Harvard University, Cambridge, MA 02138, USA; 13 Centre National de la Recherche Scientifique (CNRS) Unité Mixte de Recherche (UMR) 5199 PACEA (De la Préhistoire à l'actuel: Culture, Environnement et Anthropologie), Université de Bordeaux, Bâtiment B18, Allée Geoffroy Saint Hilaire, CS 50023, F-33615 Pessac CEDEX, France; 14 Senter for Fremragende Forskning (SFF) Centre for Early Sapiens Behaviour (SapienCE), University of Bergen, Øysteinsgate 3, Postboks 7805, 5020, Bergen, Norway; 15 Department of Anthropology, Harvard University, Cambridge, MA 02138, USA; 16 Geography, King’s College London, Strand, London, WC2R 2LS, UK; 17 Department of Anthropology, Center for Advanced Study of Hominid Paleobiology, The George Washington University, 2110 G Street North West, Washington, DC 20052, USA; 18 Department of Archaeology, University of Exeter, Exeter, UK; 19 Department of Genetics, University of Cambridge, Downing Street, Cambridge CB2 3EH, UK; 20 Wellcome Trust Sanger Institute, Wellcome Trust Genome Campus, Hinxton, UK; 21 Department of Anthropology and the Genome Center, University of California, Davis, CA 95616 USA; 22 Department of Earth and Environmental Sciences, Columbia University, Lamont-Doherty Earth Observatory, 61 Route 9 West, Palisades, NY 10964-1000, USA; 23 Department of Anthropology, Emory University, 1557 Dickey Drive, Atlanta, GA 30322, USA; 24 Laboratoire Évolution & Diversité Biologique (EDB UMR 5174), Université de Toulouse Midi-Pyrénées, CNRS, IRD, UPS. 118 route de Narbonne, Bat 4R1, 31062 Toulouse cedex 9, France; 25 Instituto Gulbenkian de Ciência, P-2780-156, Oeiras, Portugal

**Keywords:** human evolution, evolutionary genetics, paleoanthropology, paleoecology, Middle Stone Age, African origins

## Abstract

We challenge the view that our species, *Homo sapiens*, evolved within a single population and/or region of Africa. The chronology and physical diversity of Pleistocene human fossils suggest that morphologically varied populations pertaining to the *H. sapiens* clade lived throughout Africa. Similarly, the African archaeological record demonstrates the polycentric origin and persistence of regionally distinct Pleistocene material culture in a variety of paleoecological settings. Genetic studies also indicate that present-day population structure within Africa extends to deep times, paralleling a paleoenvironmental record of shifting and fractured habitable zones. We argue that these fields support an emerging view of a highly structured African prehistory that should be considered in human evolutionary inferences, prompting new interpretations, questions, and interdisciplinary research directions.

## Highlights

The view that *Homo sapiens* evolved from a single region/population within Africa has been given primacy in studies of human evolution.

However, developments across multiple fields show that relevant data are no longer consistent with this view.

We argue instead that *Homo sapiens* evolved within a set of interlinked groups living across Africa, whose connectivity changed through time.

Genetic models therefore need to incorporate a more complex view of ancient migration and divergence in Africa.

We summarize this new framework emphasizing population structure, outline how this changes our understanding of human evolution, and identify new research directions.

## A Different View of African Origins

The lineage of *Homo sapiens* probably originated in Africa at least ∼500 thousand years ago (ka) [1], and the earliest observed morphological manifestations of this clade appeared by ∼300 ka [2]. Early *H. sapiens* fossils do not demonstrate a simple linear progression towards contemporary human morphology. Instead, putative early *H. sapiens* remains exhibit remarkable morphological diversity and geographical spread. Together with recent archaeological and genetic lines of evidence, these data are consistent with the view that our species originated and diversified within strongly subdivided (i.e., structured) populations, probably living across Africa, that were connected by sporadic gene flow 1, 3, 4, 5, 6, 7, 8. This concept of ‘African multiregionalism’ [1] may also include hybridization between *H. sapiens* and more divergent **hominins** (see Glossary) living in different regions 1, 9, 10, 11, 12. Crucially, such population subdivisions may have been shaped and sustained by shifts in ecological boundaries 7, 13, 14, challenging the view that our species was endemic to a single region or habitat, and implying an often underacknowledged complexity to our African origins.

In this paper we examine and synthesize fossil, archaeological, genetic, and paleoenvironmental data to refine our understanding of the time-depth, character, and maintenance of Pleistocene population structure. In doing so, we attempt to separate data from inference to stress that using models of population structure fundamentally changes interpretations of recent human evolution.

## The Morphological Diversity and Spread of the *Homo sapiens* Clade

The constellation of morphological features characterizing *H. sapiens* is debated. This has strongly impacted on interpretations of recent human origins by variably including or excluding different fossils from interpretative analyses. For example, different morphological criteria and analytical methods have been used to support both a gradual, mosaic-like process of modernization of our species or, conversely, a punctuated speciation (e.g., [1]).

**Figure 1:**
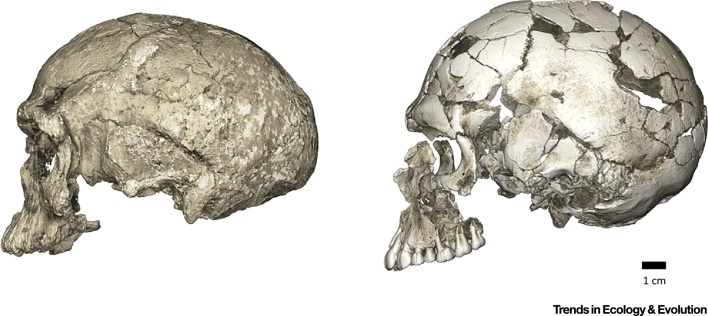
Evolutionary Changes of Braincase Shape from an Elongated to a Globular Shape. The latter evolves within the *H. sapiens* lineage via an expansion of the cerebellum and bulging of the parietal. (Left) Micro-computerized tomography scan of Jebel Irhoud 1 (∼300 ka, North Africa). (Right) Qafzeh 9 (∼95 ka, the Levant).

Extant human crania are characterized by a combination of features that distinguish us from our fossil relatives and ancestors, such as a small and gracile face, a chin, and a globular braincase. However, these typical modern human features emerge in a mosaic-like fashion within the *H. sapiens* clade. The oldest currently recognized members of the *H. sapiens* clade, from Jebel Irhoud in North Africa, have a facial morphology very similar to extant *H. sapiens*, as well as endocranial volumes that fall within the contemporary range of variation [2]. However, their braincase shapes are elongated rather than globular, suggesting that distinctive features of brain shape, and possibly brain function, evolved within *H. sapiens* 2, 5 (Figure 1). Other early *H. sapiens* fossils from Florisbad in South Africa (∼260 ka), Omo Kibish (∼195 ka) and Herto (∼160 ka), both in Ethiopia, are morphologically diverse 1, 16. This diversity has led some researchers to propose that fossils such as Jebel Irhoud and Florisbad actually represent a more primitive species called ‘*H. helmei*’, using the binomen given to the Florisbad partial cranium in 1935 17, 18. In a similar vein, the fossil crania from Herto [19], which combine a relatively globular braincase with a robust occipital and large face, were described as the subspecies *H. sapiens idaltu* because they fall outside the variation of recent humans.

However, we view *H. sapiens* as an evolving lineage with deep African roots, and therefore prefer to recognize such fossils as part of the diversity shown by early members of the *H. sapiens* clade. The full suite of cranial features characterizing contemporary humans does not appear until fairly recently, between about ∼100–40 ka [20]. The character and chronology of early *H. sapiens* fossils, together with their geographic distribution across Africa, suggests that evolution may at times have progressed independently in different regions, in populations that were often semi-isolated for millennia by distance and/or ecological barriers, such as hyperarid regions or tropical forests.

Further insights into the geographic extent and potential habitat diversity of early *H. sapiens* populations can be gained from more recent forager populations in Africa, which were also strongly structured. For example, **Later Stone Age** (LSA) human remains highlight both the retention of ‘archaic’ traits and the maintenance of considerable morphological diversity into the terminal Pleistocene 11, 21. In the **Holocene**, the skeletal record becomes much richer, but there remains considerable spatial variation in morphology. Variation between populations in different regions and environments of Africa may have been shaped by isolation-by-distance and local environmental adaptations 22, 23, 24, 25, 26. For example, challenging environments (e.g., deserts, rainforest) and isolation have likely played a significant role in shaping the population structure of Holocene African foragers and isolated hunter-gatherers across the tropics 25, 27.

Ultimately, the processes underlying the emergence of any ‘package’ of derived features diagnostic of early *H. sapiens* anatomy remain incompletely understood. However, the data do not seem to be consistent with the long-held view that human ancestry is derived predominantly from a single African region hosting a **panmictic** population. Instead, *H. sapiens* likely descended from a shifting structured population (i.e., a set of interlinked groups whose connectivity changed through time), each exhibiting different characteristics of anatomical ‘modernity’. The discovery that the primitive-looking *H. naledi* dates to between ∼335 ka and 236 ka [28], and that the Broken Hill 1 ***Homo heidelbergensis*** skull may date to ∼300–125 ka [29], also shows that other hominin species in Africa coexisted with *H. sapiens*, raising the possibility of African archaic interbreeding. Future research should attempt to determine which features evolved before the appearance of our species and which primarily developed within the evolutionary history of our species. Another key area concerns understanding the extent to which different processes shaped observed changes. For example, the narrowing of the pelvis may reflect different processes including neutral genetic drift, adaptation to ecological variation, and life-history variation.

## A Pan-African Cultural Patchwork

Across Africa, the virtual abandonment of handheld **large cutting tools** such as handaxes, and an increased emphasis on **prepared core technologies** and **hafting**, marked a profound technological reconfiguration of hominin material culture. These technological changes, which define the transition to the **Middle Stone Age** (MSA), seem to have occurred across Africa at a broadly similar time; for example at ∼300 ka both at Jebel Irhoud, where they are found with early *H. sapiens* fossils [16], and at Olorgesailie in East Africa [30], and at ∼280 ka in southern Africa at Florisbad [31]. Currently, the earliest dates in West Africa are younger, at ∼180 ka, but the region remains very poorly characterized [32]. The MSA is associated with *H. sapiens* fossils, but both *H. naledi* and *H. heidelbergensis* probably persisted into the late **Middle Pleistocene**.

**Figure 2:**
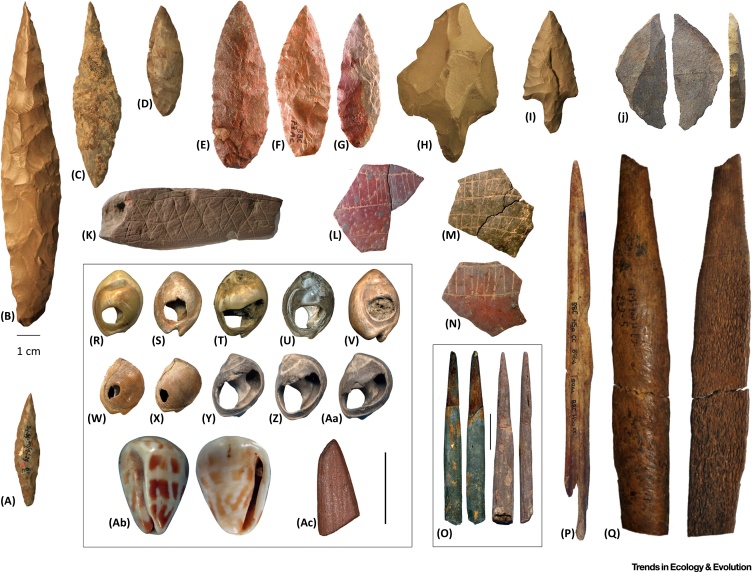
Middle Stone Age Cultural Artefacts. (A–D) Bifacial foliates from northern Africa (A, Mugharet el Aliya; B–D, Adrar Bous). (E–G) Bifacial foliates from southern Africa (Blombos Cave). (H,I) Tanged tools from northern Africa. (J) Segmented piece bearing mastic residue from southern Africa (Sibudu). (K) Engraved ochre fragment (Blombos Cave). (L–N) Engraved ostrich eggshell fragments from southern Africa (Diepkloof). (O,P) Bone points from southern Africa (Sibudu and Blombos Cave, respectively). (Q) Bone point from northern Africa (El Mnasra). (R–V) Perforated *Trivia gibbosula* shells from northern Africa (R,S, Grotte de Pigeons; T–V, Rhafas, Ifri n’Ammar, and Oued Djebbana, respectively). (W–Aa) Perforated *Nassarius kraussianus* shells from Blombos Cave. (Ab) *Conus ebraeus* shell bead (Conus 2) from southern Africa (Border Cave). (Ac) Ochre fragment shaped by grinding from southern Africa (Blombos Cave). All scales are 1 cm. Boxed items indicate rescaled artefacts. Images reproduced, with permission, from (A–D, H, I) The Stone Age Institute; (E–G, J–P, Ac) from [35]; (Q) from [47]; and (R–Ab) from 35, 47, 48.

Clear regionally distinctive material culture styles, typically involving complex stone tools, first emerged within the MSA. For example, the Central African MSA includes heavy-duty axes, bifacial lanceolates, backed flakes and blades, picks and segments, probably from at least the late Middle Pleistocene [33]. In the **Late Pleistocene**, grassland and savannah expansion in North Africa led to dense human occupation associated with specific regional technological features such as **tanged implements** (Figure 2) [34]. At approximately the same time there was an emergence of comparably distinctive industries in parts of southern Africa. As in North Africa, some of these industries are also associated with other aspects of complex material culture such as ochre, bone tools, shell beads, and abstract engravings (Figure 2) [35].

Such regionalization is typically linked with the emergence of ‘modern’ cognition. However, it arguably also reflects the interaction between demographic variables (e.g., increased population density) 36, 37, 38 and the learned traditions of long-lived regional subpopulations or demes (Figure 2). For example, northern and southern Africa, apart from being geographically distant, were also separated by environmental factors as a consequence of the expansion and contraction of forests in equatorial Africa, synchronous with amelioration in northern Africa. Other factors, such as habitat variability and adaptation to local environmental conditions, are also likely to play some role in material culture diversification.

Although geographical differences are clear at the continental scale, localized spatial patterning is harder to discern. Similarities between regions may have been produced by occasional contact or by convergent adaptation to common environmental conditions. In East Africa, for example, although there is certainly some variation, there appears to be underlying continuity in material culture throughout much of the MSA (e.g., [39]). In many regions, ‘generic’ MSA assemblages that do not carry an obvious signal of regionalization are common [40]. In a cognitive model, these differences suggest that not all these early populations manifested a ‘modern mind’. However, such assemblages are augmented by shifting frequencies of tool types that appear to be spatially or temporally indicative, and likely reflect demographic factors. In some parts of Africa, the full suite of generalized MSA characteristics continues largely unchanged until the Pleistocene/Holocene boundary [41], matching the morphological patterns, and suggesting that the end of the MSA may have been as structured and mosaic-like as its beginnings. This view has support from LSA material culture. Despite superficial similarities in LSA lithic miniaturization, the cultural record shows continued differentiation and derivation into the Holocene, supporting the biological evidence for variable population dynamics that did not result in wide-scale homogenization [42].

The reasons for, and therefore implications of, the geographic and temporal structuring of MSA cultural diversity are still poorly characterized and likely reflect several processes. These include adaptations to different environments [43]. Long-term, large-scale population separation may also have been the norm for much of Pleistocene Africa (Box 1; i.e., isolation by distance and isolation by habitat, representing null models to be rejected). Rare and spatially explicit models exploring Pleistocene technological innovations have also linked cultural complexity with variation in regional patterns of population growth, mobility, and connectedness (e.g., 36, 44, 45), supported by evidence of long-distance transfer of stone raw material (e.g., [46]).

**Figure I:**
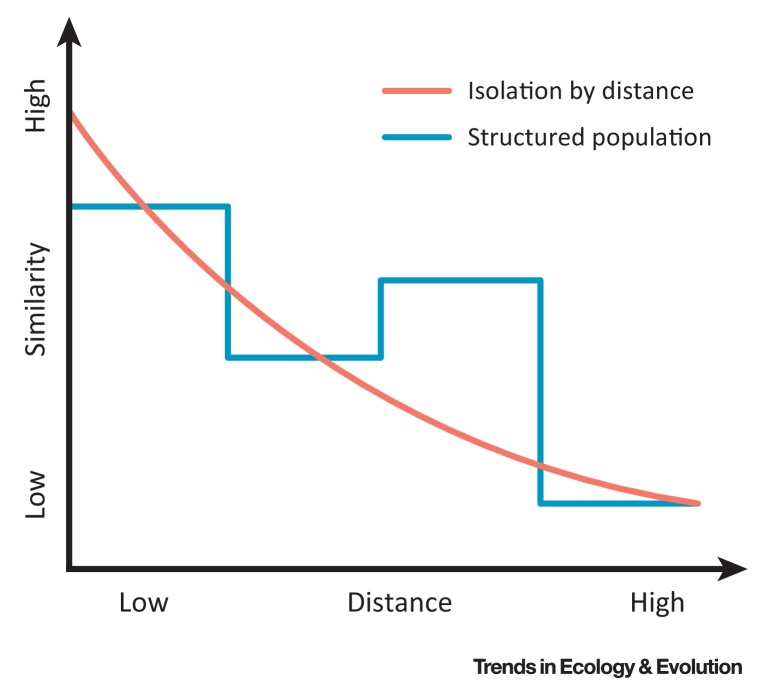
Simple IBD Model with Cultural Data. Note that similarity can increase with distance under some circumstances, for example when similar habitats are separated by considerable distances, with areas of different habitat types being located between them.

Box 1 **Isolation by Distance (IBD)**

IBD is the expectation that genetic differences correlate positively geographic distances as a consequence of the fact that mating is more likely to occur at shorter than longer distances. This concept, although well established in genetics, is rarely applied to Pleistocene archaeological and human fossil material, despite its potential value as a null model for observed cultural or morphological differences between materials from different sites [49].

Because archaeological problems are concerned with identifying the processes that generate observed patterns of cultural variation over time and space, the lack of equivalent null models is particularly problematic. Processes generating cultural variation constitute a complex balance between patterns of inherited knowledge, local innovation, modes of cultural transmission, local adaptation, and shifting population dynamics (e.g., population size, density, or mobility [36]). Without null models of ‘cultural similarity’ as a baseline, it is difficult to escape simplistic, narrative inferences about the past. Similarly, human fossil data can be interpreted in several ways depending on the taxonomy employed, but spatial variation is likely to relate to the same factors that influence genetic similarities between regional populations [50].

In an archeological context, the expectation of an IBD model is that cultural similarity will decrease with distance, with a degree of spatial autocorrelation (e.g., [51]). This expectation represents the simplest explanation of the observed variation. If this null model does not provide an adequate explanation of the data, more complex models can be invoked to explain patterns observed either in the residuals from the null model or in the raw data as a whole. For example, a more complex population structure can be theoretically differentiated from an IBD model if patterns of spatial autocorrelation are discrete rather than continuous (Figure I), pointing towards the formation of distinct biological or cultural clusters which may correlate with other features (e.g., genetic, morphological, or environmental). Many factors could promote the formation of such clusters, including assortativity by cultural similarity, or conformity to local norms of, for example, tool production.

Alt-text: Box 1

Major new archaeological research directions should include: (i) unraveling the relative contributions of different African regions/habitats to recent human evolution; (ii) understanding shifting patterns of population structure through the differential appearance, expansion, contraction, and disappearance of regionally distinct artefact forms (Box 1); and (iii) exploiting the growing interface between archaeology, ecology, morphology, and genetics to explore the extent to which material culture patterning is coupled or decoupled from these associated (but potentially independent) axes.

## Why Genetic Models Must Incorporate a More Complex View of Ancient Migration and Divergence in Africa

The starting point for most genetic studies of human origins has been to investigate the depth of present-day diversity between and within African populations. Most studies have used simple ‘tree-like’ demographic models to infer population split times, neglecting or simplifying population structure, even if sometimes considering a degree of gene flow between branches (Box 2). Such studies have produced a variety of split-time estimates, with the KhoeSan populations of southern Africa, who retain the greatest levels of genetic diversity among human populations today, comprising one branch of the deepest divergence inferred, at 150–300 ka 52, 53, 54, 55, 56, 57, 58. Some authors have interpreted this, in conjunction with a gradient of south to north decreasing genetic diversity within Africa, as favoring a single-origin model for modern humans with a locus in southern Africa rather than in eastern Africa 59, 60. Variation in inferred split times reflects a variety of different methodologies, model assumptions, and data sources, with a general trend for more recent analyses to infer older dates. In addition to ancient gene flow and structure, more recent population movements within Africa, such as the expansion of Bantu-speaking peoples from West Africa at 2–1.5 ka 52, 61, will have obscured signatures of older demographic processes, as will have episodes of ‘back-to-Africa’ migration from Europe and southwest Asia into several regions of the continent 60, 62, 63, 64, 65.

Box 2 **Modeling Population Structure**

Population genetic modeling for demographic inference often assumes panmixia, wherein all individuals in a population have an equal chance of mating with one another. In such cases there is often an implicit assumption that panmixia occurs at the whole-species level. However, real populations rarely meet this condition, owing to the existence of spatial structure at the species level or other stratification of individuals within the population. Population structure and its consequences for genetic data can be modeled in various ways. For example, Wright’s *n*-island model assumes that populations are subdivided into *n* different islands/demes that are connected through gene flow. This simple model assumes that all demes are panmictic, have the same size, and exchange genes with all other islands at the same rate. Other models include tree models in which an ancestral population splits into two or more populations that may themselves later split. The *n*-island model ignores space (i.e., differing levels of connectivity between populations), whereas in tree models geographic location may be implicit but is not explicitly modeled. For example, two populations that are geographically close may be assumed to share a more recent splitting event than populations that are more distant. Other models (e.g., stepping stone) may incorporate geographic space explicitly by connecting demes via gene flow only with their spatial neighbors.

Another distinction among model types is whether they incorporate change over time. The stepping-stone and *n*-island models have no temporal aspect. Conversely, tree models may generate very different results depending on the timing of sampling in relation to the splitting events and gene flow between populations. More complex models allow demographic expansions or contractions in space, often based on simulations 71, 72. Finally, the class of ‘meta-population models’ include demes of variable sizes that can be connected by gene flow and colonization events. The population in each deme can also become extinct and be recolonized by individuals coming from one or several other populations.

This diversity in range of structured population demographic models, with varying levels of complexity, leads to some arbitrariness in which models are chosen. Because we know that the human past was complex, it is often assumed that more complex models of that past are more realistic. However, more complexity means more parameters and more ways for a model to differ from reality. This means that unless informed *a priori* by secure information, or fitted to substantial quantities of conditioning data, more complex models can be more wrong, not more realistic. The past two decades have also seen the development of many different inference methodologies, the results of which are often difficult to compare. This is because they often make different assumptions (tree splitting versus spatial distribution), explain different aspects of the data (**allele-frequency spectrum**, AFS; versus IICR), and can be computationally demanding – challenging interpretation, explanatory power, and validation, respectively. Models are valuable tools for understanding and interpreting data, but we should not be surprised if a single family of models is unable to explain all patterns of human genetic diversity.

Alt-text: Box 2

Models incorporating more complex population structure can be considerably more parameter-rich and therefore more difficult to test computationally, particularly with limited data. However, they do offer a more generalized and flexible view of past demography – one that can accommodate, but not be limited to, more traditional population tree models. Furthermore, the increased availability of genomic data and developments in analytical methodologies now permit inference under more complex and realistic models. These developments have shown that structure cannot be neglected, and can cause patterns in genetic data that are similar to those generated by other forms of demographic change (e.g., 66, 67). For example, inferred changes in **effective population size** (*N*_e_) may result from changes in connectivity between ancient populations rather than from, or in addition to, changes in census population size 7, 68. Indeed, the relationship between inferred *N*_e_ and census population size is not straightforward, and may even be counterintuitive when structure exists 7, 66, 68. The geographical scale at which population genetic structure may have existed is also difficult to infer. For example, one recent genomic study showed substantial structure between pre-agricultural human populations separated by only tens or hundreds of kilometers [69]. These insights challenge the view that the early prehistory of our species can be well approximated by population growth within a single lineage [70].

**Figure 3:**
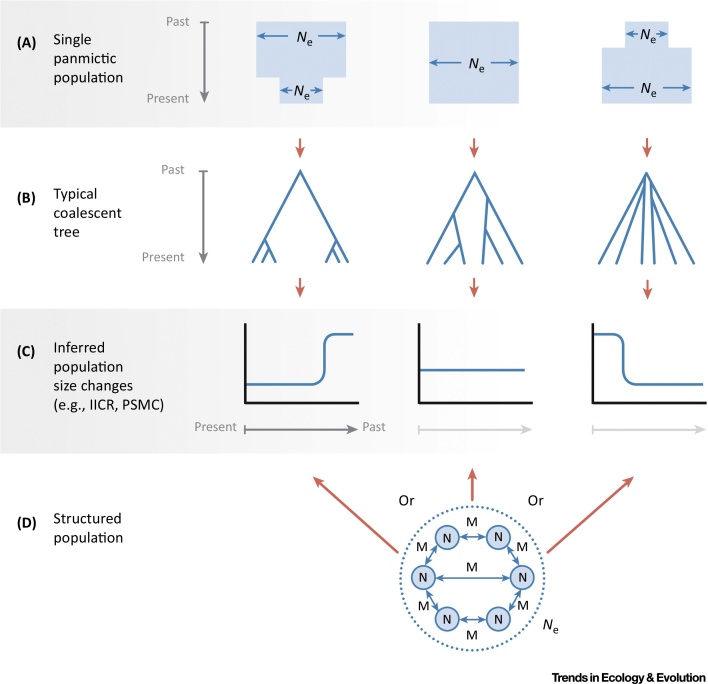
Inferring Population Size Change in Unstructured and Structured Populations For a Figure360 author presentation of Figure 3, see the figure legend at https://doi.org/10.1016/j.tree.2018.05.005 Figure360: an author presentation of Figure 3Figure 3 The probability, or expected rate, of coalescence of lineages in a single panmictic population is inversely proportional to the population size at the time. Different panmictic population size histories (A) therefore shape the temporal distribution of coalescent events (B). When estimated for many regions of the genome, the temporal distribution of these nodes can be used to estimate the **instantaneous inverse coalescence rate** (IICR), which in a single panmictic population is a direct proxy for the population size (C). Software such as the **pairwise sequentially Markovian coalescent** (PSMC) or the multiple sequentially Markovian coalescent (MSMC) can be used to estimate the IICR/population size change in the past. However, when data are sampled from a structured meta-population consisting of subpopulations connected by migration (D), changes in migration through time, and/or in sampling, can generate any IICR-inferred population size history without any actual change in the meta-population size (*N*_e_).

Although modern genomic data have been shaped by, and thus contain large amounts of information on, past demography, these data can be explained by many different models of population history (equifinality). Moreover, all such models are necessarily abstractions and simplifications of the true population histories, and the discrepancies involved may be particularly problematic for certain questions about the past (mis-specification). This means that structure can be difficult to unambiguously detect, and even harder to reconstruct. For example, several studies on African populations have identified genes with coalescence times on the order of 1 million years, which could be interpreted as indicating admixture with archaic hominins 9, 10, 73. However, even in a single population some very old coalescence times (>1 My) are expected for humans (Figure 3), and therefore inferences based on the tail of the distribution of coalescence times, which are particularly sensitive to model mis-specification, need to be interpreted with caution. Indeed, several authors have argued that deep coalescence times are compatible with a single human lineage in Africa with deep population structure 7, 68, 74.

Ancient DNA (aDNA) data can provide additional resolution, and studies on Holocene individuals recently revealed extensive structure and migratory activity during that period 8, 75. Ancient Pleistocene-aged aDNA would be more informative, but is difficult to obtain because tropical environments are mostly unfavorable for DNA preservation. However, a recent study showed that Late Pleistocene aDNA can be retrieved in some African regions [65]. These studies demonstrate that inferences from patterns of human genomic diversity need to consider fluctuating population structure over long periods, in addition to the range of panmictic African population origin models.

## Environmental and Ecological Drivers of Population Structure

**Figure 4:**
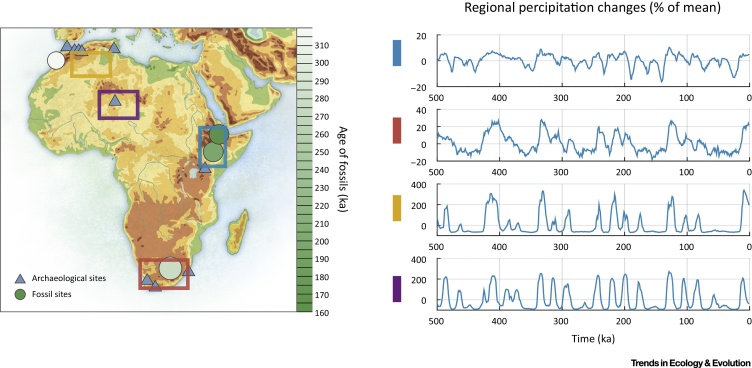
Middle and Late Pleistocene African Environmental Variability in Space and Time. (Left) Map of Africa with key archaeological and fossil sites discussed in the text. Colored boxes indicate averaged regions for simulated precipitation changes from the transient glacial/interglacial LOVECLIM climate model experiment [81]. (Right) Precipitation changes (%) relative to the long-term 784 thousand year mean in the key regions highlighted in left panel, as simulated by transient 784 thousand year-long LOVECLIM climate model simulation [81]. From top to bottom the regions are eastern equatorial Africa, southern Africa, northwestern Africa, and the central Sahara region.

The genetic, fossil, and archaeological data discussed above indicate that *H. sapiens* evolved in highly structured populations, probably across many regions of Africa. Elucidating the degree of and mechanisms underlying population structure will require consideration of Middle and Late Pleistocene environmental variability in both space and time (e.g., [13]) (Figure 4). **Refugia** have been highlighted as key catalysts of evolutionary change [76], and certainly would have generated population structure. Nevertheless, some regions acting as ‘backwaters’ and isolated habitat islands may also have been central in the persistence of relict populations. Research has emphasized broad asynchronous environmental changes in different African regions (e.g., 13, 77). The northern and southern tips of the continent are most strongly affected by winter westerly precipitation, variation in which is largely driven by changes in Atlantic Ocean circulation. However, most of Africa experiences monsoonal rainfall associated with the **intertropical convergence zone** (ITCZ), the strength and location of which varies according to changes in insolation that are driven primarily by **precessional** aspects of **Milankovitch forcing**. Consequently, parts of tropical Africa that are currently humid likely experienced numerous episodes of extreme aridity in the past 78, 79. At the same time that the monsoon migrated northwards, the Sahara contracted, and networks of lakes and rivers expanded across much of north Africa 80, 81, 82, with matching conditions in parts of southwest Asia. Finer-scale shifts in the monsoon are also evident. For example, in West Africa the extent of savannah and forested areas is strongly affected by small changes in patterns of rainfall 83, 84.

Climate therefore varied greatly, and periods of relatively increased aridity or humidity were asynchronous across Africa. Crucially, these factors are major drivers of faunal population structure and speciation 85, 86, illustrated by numerous sub-Saharan animals exhibiting similar phylogenetic patterns in their distribution. For example, Bertola and colleagues [87] show that dozens of species exhibit distinct populations in the two major evolutionary realms of west/central Africa and east/southern Africa. Many also show a further subdivision between east Africa and southern Africa, signifying important refuges in these three regions. These species occupy a range of trophic levels, suggesting that climate affected whole ecological communities.

Therefore, faunal speciation largely appears to have been catalyzed by climate-driven habitat fragmentation and interaction between different biomes over time. This provides insights into how human population structure could have been maintained over significant timescales and geographic areas of Africa. In Africa, the concept of ‘refugium networks’ has been specifically implicated in Pleistocene human population subdivisions and expansions [18], and as a result such regions are of major evolutionary interest. Although fragmentation of suitable habitat has been highlighted as a major driver of population structure over and above isolation-by-distance (Box 1), isolation-by-habitat can also play an important role in animal [84] and probably also human population structure 14, 88.

Major challenges remain in integrating fossil, archaeological, and genetic lines of evidence into paleoenvironmental and paleoecological contexts (but see [3] and [72] for diverse attempts to do so). Currently, the chronology of much of the paleoanthropological record remains too coarse to allow any firm conclusions to be drawn about the role of environmental changes. Promising new avenues of research include genomic analyses of fauna, including the identification of commensal species and reconstructions of human habitats through stable-isotope analyses.

## Concluding Remarks: Moving Forwards

Available morphological, archaeological, genetic, and paleoenvironmental data indicate that the subdivision of Middle and Late Pleistocene African human populations drove the mosaic-like emergence and evolution of derived *H. sapiens* morphology. Reproductively semi-isolated populations adapted to local ecologies alongside drift. Such population isolation was likely facilitated by small population sizes. Thus, as with other fauna, gene flow should not be assumed to have been constant through time, or to have occurred at the same rate within and between different regions. Across the large timescales of the Middle and Late Pleistocene, with their strong climatic fluctuations, the number of intermediate populations connecting different regions is also likely to have fluctuated considerably.

Several major unanswered questions flow from this reorientation of recent human origins (see Outstanding Questions). Did key diagnostic morphological characteristics emerge in one region and become elaborated with subsequent dispersals? Or did the transition from ‘archaic’ to ‘modern’ – whether indicated by morphology or material culture – occur gradually, and in a mosaic-like fashion across the continent? If this was the case, did African archaic hybridization also play a role? How does the existing evidence for structure affect our understanding of the history of population size changes and dispersals? Similarly, we have no firm grasp on the concordance that might exist between morphological and cultural structuring. Regional cultural signatures are apparent, raising the possibility that spatially distinct forms of material culture reflect similar patterns of population isolation and aggregation. Filling in these knowledge gaps requires us to reconsider paleoanthropological species concepts which are challenged by the view of deep population structure with sporadic gene flow/admixture.

Ultimately, reconstructing the demographic history of human populations in its full complexity is beyond the power of population genomics alone, necessitating an interdisciplinary approach. In the past this has been achieved by geneticists working with archaeologists and paleoanthropologists to define a narrow set of simplified hypotheses whose genetic outcomes can be compared to identify the models that best explain the data. While such an approach has met with considerable success, a more complete picture will require integrating different data types (genetic, fossil, material culture, paleoclimate and paleoecological data) using the same or analogous models of population structure, size change, and dispersal. This represents a major challenge for ancestral demographic inference over the coming years.

Fully characterizing the nature of this apparent ‘African multiregionalism’ also requires rejecting numerous longstanding, if implicit, assumptions, and formulating new questions. For example, the chronological lag between genetic estimates of population divergence times and morphological changes in the fossil record is not well understood, and should not be assumed to be short – particularly because inferences from genetic data are profoundly influenced by the models or families of models used. For instance, the estimates of population split times that are sometimes published may become less appropriate or relevant in our understanding of human evolution if models of spatial structure are to be used.

Similarly, while a globular braincase does seem to represent a synapomorphy of extant *H. sapiens*, can it be effectively characterized for application to the fossil record? We emphasize that *H. sapiens* is a lineage with deep and likely diverse African roots that challenge our use of terms such as ‘archaic *H. sapiens*’ and ‘anatomically modern humans’. Unless they can be operationalized with more clearly defined traits, such categories will have declining value. Diagnostics of *H. sapiens* must reflect trajectories of evolution rather than static views of our species – which has changed, and continues to change, at various scales.

The next decade of research will be crucial to resolving these emerging research themes. Contemporary human genomes are now available from across the African continent, together with an increasing number of ancient genomes. Our understanding of paleoecology is also improving thanks to biogeographic reconstructions premised on the genomes of African fauna. Paleoclimate reconstructions are increasingly precise, with rapidly growing proxy data and better models covering key periods. Finally, the expansion of paleoanthropological investigations into neglected areas of Africa will undoubtedly reveal new data that will significantly refine the parameters of recent human evolution.

Outstanding Questions In the conventional view, *H. sapiens* emerged in one region and/or population of Africa. Instead, new data suggest that a variety of transitional human groups, with a mosaic of primitive and derived features, may have lived over an extensive area from Morocco to South Africa between >300 ka and 12 ka.

Three outstanding questions emerge from this view. First, within the African ‘multiregional’ paradigm, which species best fits as the ancestor(s) of *H. sapiens*? Many aspects of the delicate *H. sapiens* facial shape may not be derived but instead be primitive retentions from an ancestor with a generalized facial shape. It therefore seems possible that *H. sapiens* did not evolve from the African forms of *H. heidelbergensis* (as represented, e.g., by the Bodo skull from Ethiopia, and Broken Hill from Zambia), but from a more primitive *H. antecessor* or *H. erectus*-like ancestor, beginning at ∼0.5 Ma 1, 2. However, hybridization during the inception of this process is also a possibility. Resolving the speciation of *H. sapiens* and the character of ancestral populations represents a crucial first step in understanding the emergence of the morphological features that diagnose our species during the later Middle Pleistocene.

Second, how many populations, environments, and geographical areas of Africa played a role in the origins of *H. sapiens*? Did adjoining areas of western Asia also play a part? It seems possible that early humans followed the same ecological partitioning and subspeciation patterns that are seen among continentally distributed African mammals, many of which emerged at the same time as the genus *Homo*. The Sahara may have played a particularly important role in this respect. Other areas, such as regions of forest, may also have supported populations who remained semi-isolated from those in grasslands and savannahs. Addressing the challenges of research in deserts and rainforests will be difficult, but is likely to be rewarding.

Finally, were some of our anatomical traits inherited from transitional African forms before they became extinct? The range of dates for *H. naledi* and *H. heidelbergensis* confirms the late survival of at least two archaic species in Africa. The size and environmental diversity of Africa, particularly the poorly investigated forested regions, may have permitted the late survival of more archaic species as well as of early forms of *H. sapiens*. These discoveries have fuelled speculations that *H. sapiens* may have interbred with archaic species in Africa itself. Distinguishing admixture between species from the reintegration of diverse *H. sapiens* lineages represents a major challenge, with significant taxonomic implications.

## Glossary

**Allele-frequency spectrum (AFS)**: also known as or site-frequency spectrum (SFS), AFS is a histogram representing the frequencies of the alleles from multiple loci (e.g., SNPs). Assumes that loci are biallelic, and is used as a summary of genomic data for demographic inference.
**Archaic hominin**: umbrella term for any of a broad group of non-*Homo sapiens* humans, such as the Neanderthals or *Homo heidelbergensis* (see below). Archaic features within our species refers to the retention of particular traits typically associated with archaic hominins, such as an elongated (versus a modern globular) braincase.
**Core-and-flake technology**: stone tool technology focused on the removal of flakes as desired products from a block of raw material, which is ultimately discarded as waste. This stands in contrast to technology involving the shaping of raw material into a product (e.g., a handaxe), where the shaping flakes are discarded as waste.
**Effective population size (*N*_e_)**: a measure of genetic drift, and indirectly of some measure of population genetic diversity. *N*_e_ represents the size of the ideal population that would have the same drift properties as the real population. Depending on the properties of the genetic data in which we are interested, different effective sizes may be defined. In an ideal population, the different *N*_e_ values should be the same, but in real populations this may not be the case. *N*_e_ can be seen as the number of individuals actually contributing to the next generation, as opposed to the total number of individuals in a population (which is usually much larger).
**Hafting**: the process of attaching a stone or a bone tool to a haft, typically but not always constructed from wood, either through adhesives (gum, resin), bindings, or both.
**Holocene**: the recent geological epoch which began at around ∼11.7 ka and which is associated with the current warm period.
***Homo heidelbergensis***: originally named for the Mauer mandible in 1908. Some researchers (e.g., [15]) have argued that this species was widespread in Eurasia and Africa during the Middle Pleistocene. Others prefer to distinguish the African samples as *H. rhodesiensis*, based on the Broken Hill cranium found in 1921.
**Instantaneous inverse coalescence rate (IICR)**: the IICR is a time- and sample-dependent parameter. In a panmictic population, the IICR is proportional to *N*_e_. In structured populations, the IICR can be strongly disconnected from changes in *N*_e_.
**Intertropical convergence zone (ITCZ)**: a zone near the equator where the northern and southern air masses converge, typically producing low atmospheric pressure and considerable convective rainfall.
**Large cutting tools**: blocks of stone that were shaped into large handheld tools for cutting functions, such as stone axes. The flakes chipped off the stone block are referred to as thinning flakes and were typically waste products.
**Last Interglacial**: the previous interglacial to the current one; this began at ∼125 ka and ended at ∼109 ka. Interglacials are warm periods characterized by receding ice sheets in the higher latitudes and increased humidity in the mid-latitude arid belt.
**Late Pleistocene**: the final part of the Pleistocene geological epoch, beginning with the **Last Interglacial** at ∼125 ka and lasting until the beginning of the Holocene at ∼11.7 ka.
**Later Stone Age (LSA)**: a cultural-technological phase in Africa dating broadly from ∼60 ka to 5 ka. While highly variable and poorly defined, the LSA is characterized by a focus on the production of small tools, such as blades and bladelets, and geometric microliths.
**Middle Pleistocene**: the middle part of the Pleistocene geological epoch, beginning at ∼781 ka and ending with the beginning of the Last Interglacial at ∼125 ka ago. The Middle Pleistocene is associated with the emergence of *Homo sapiens* and *Homo neanderthalensis* at >300 ka.
**Middle Stone Age (MSA)**: a cultural-technological period in Africa characterized by the widespread use of **core-and-flake technologies**, which were often hafted, at around ∼300 ka. The MSA gave way to the LSA over a protracted period broadly between ∼60 ka and 20 ka which featured the replacement of classic core-and-flake technologies with small and often geometric microlithic tools. The MSA is in general similar to the contemporaneous Middle Paleolithic of Eurasia.
**Milankovitch forcing**: the effect on climate of slow changes in the tilt of the Earth’s axis and shape of its orbit that change the amount and distribution of sunlight reaching the Earth.
**Pairwise sequentially Markovian coalescent (PSMC)**: a method (and associated program) producing a plot showing changes in *N*_e_ from a single diploid (or two haploid) genome(s) [16]. The PSMC actually estimates the IICR and has been used on many species.
**Panmixia**: random patterns of mating within a population.
**Precession**: climatically relevant change of the Earth’s rotation with a period of 26 000 years. In combination with the slow rotation of Earth’s elliptical orbit around the Sun, the main solar incoming radiation changes vary with a periodicity of 21 000 years. The effect of precession on the climate system occurs preferentially for large elliptic orbits.
**Prepared core technology**: **a form of core-and-flake technology** where the core is shaped in such as a way as to predetermine the shape of subsequent flake removals. It was the dominant technology of the MSA. The most iconic form of prepared core technology is Levallois technology.
**Refugia**: regions or areas that remain consistently habitable by a species or association of species through an entire glacial and interglacial climatic cycle.
**Tanged implements**: stone tools characterized by a stem or a tang at the base of the tool, which is thought to be inserted into the socket of a wooden haft. This would allow stone spear points to be attached to wooden shafts, for example.
**Upper Paleolithic**: a cultural-technological period in Eurasia between ∼45 ka and 10 ka that is generally characterized by an emphasis on blade and bone technology, cave art, carvings, varied personal ornaments, and elaborate burials. In Europe it is typically regarded as a marker of *Homo sapiens*.
